# Evaluating large language model workflows in clinical decision support for triage and referral and diagnosis

**DOI:** 10.1038/s41746-025-01684-1

**Published:** 2025-05-09

**Authors:** Farieda Gaber, Maqsood Shaik, Fabio Allega, Agnes Julia Bilecz, Felix Busch, Kelsey Goon, Vedran Franke, Altuna Akalin

**Affiliations:** 1 Berlin Institute for Medical Systems Biology (BIMSB), Max Delbrück Center for Molecular Medicine, Berlin, Germany; 2 Department of Computer Science, Humboldt-Universität zu Berlin, Berlin, Germany; 3 Department of Radiation Oncology, Fondazione Policlinico Universitario Agostino Gemelli, Rome, Italy; 4 Department of Obstetrics and Gynecology/Section of Gynecologic Oncology, University of Chicago, Chicago, IL USA; 5 Institute for Diagnostic and Interventional Radiology, TUM School of Medicine and Health, TUM University Hospital Rechts der Isar, Technical University of Munich, Munich, Germany

**Keywords:** Computational biology and bioinformatics, Signs and symptoms, Diseases, Medical research

## Abstract

Accurate medical decision-making is critical for both patients and clinicians. Patients often struggle to interpret their symptoms, determine their severity, and select the right specialist. Simultaneously, clinicians face challenges in integrating complex patient data to make timely, accurate diagnoses. Recent advances in large language models (LLMs) offer the potential to bridge this gap by supporting decision-making for both patients and healthcare providers. In this study, we benchmark multiple LLM versions and an LLM-based workflow incorporating retrieval-augmented generation (RAG) on a curated dataset of 2000 medical cases derived from the Medical Information Mart for Intensive Care database. Our findings show that these LLMs are capable of providing personalized insights into likely diagnoses, suggesting appropriate specialists, and assessing urgent care needs. These models may also support clinicians in refining diagnoses and decision-making, offering a promising approach to improving patient outcomes and streamlining healthcare delivery.

## Introduction

Clinical decision-making is a fundamentally complex process that relies on clinicians applying their knowledge and experience^1^ while considering numerous factors and integrating vast amounts of data to assess patient symptoms, determine the severity of their condition, and choose the most appropriate next steps. This process typically involves combining information from various sources, such as symptoms, vital signs, patient medical history, and various examinations, to arrive at an accurate and timely diagnosis. The ability to correctly interpret this information and make well-founded decisions is crucial for improving patient outcomes. In a saturated healthcare system with increasing amounts and complexity of patient data, fewer healthcare professionals face the challenge of meeting increasing patient demands for fast, accurate, and personalized care. Especially in high-pressure environments like emergency departments, the fast pace and complexity of decision-making can contribute to delays or errors in triaging, diagnosis and treatment, ultimately leading to suboptimal care.

Recent advancements in large language models (LLMs) have demonstrated significant potential to transform various fields, including clinical decision-support^2,3^. While LLMs have shown promise in structured environments, such as medical licensing exams and clinical vignettes^4,5^, their application in real-world, open-ended clinical scenarios remains an emerging area of research. Powerful models could increase diagnostic accuracy, optimize triage processes and improve patient management. For example, LLMs could assist in prioritizing patients based on symptoms and vital signs, distinguishing between urgent and non-urgent cases, thereby reducing waiting times and improving care delivery. This capability is especially crucial in emergency departments (EDs), where accurate triage (level of severity of a patient’s condition) assessment is vital for patient prioritization. Errors in this process—whether under-triage (assigning lower urgency than needed) or over-triage (assigning higher urgency)— significantly impact patient outcomes and resource allocation.

Trauma systems have set the goal to minimize under triage and accept a higher rate of over triage to reduce mortality rate caused by under triage, with goals set at ≤5% and ≤35%^6^, respectively. A review of field triage performance showed 14% to 34% under triaged cases across all ages^7^, which can result in delayed treatment for patients requiring immediate care, potentially worsening their outcomes. On the other hand, over-triage rates were shown to be between 12% and 31%^7^, leading to the waste of critical resources and increased waiting times for other patients. In this context, LLMs might mitigate both under-triage and over-triage, thereby improving resource allocation and overall patient outcomes.

Beyond assisting clinicians, LLMs could help patients manage their own healthcare decisions. These models have the potential to guide patients in interpreting their symptoms, recommend appropriate specialists and determine the best course of action. However, while the capabilities of LLMs are promising, their real-world application in dynamic and unstructured clinical environments remains an area of active research and development.

While the scope of current LLM research in healthcare focuses on diagnosing specific diseases or targeting particular medical specialties, which are necessary and hold significant promise^8–14^, it misses the broader task of predicting diagnoses to support comprehensive clinical decision-making in more general, fast-paced environments. Other studies employ models that are required to choose a diagnosis from a simplified set of binary or multiple-choice options testing human competencies within particular domains^15–18^ which reduces the complexity of real-world clinical decision-making. In practice, clinicians are frequently faced with vague or unclear symptoms, incomplete information, and unlike in controlled studies, they do not have the convenience of selecting from multiple-choice options. Instead, they must rely on their clinical judgment and experience to navigate uncertainty and arrive at a diagnosis.

**Fig. 1:**
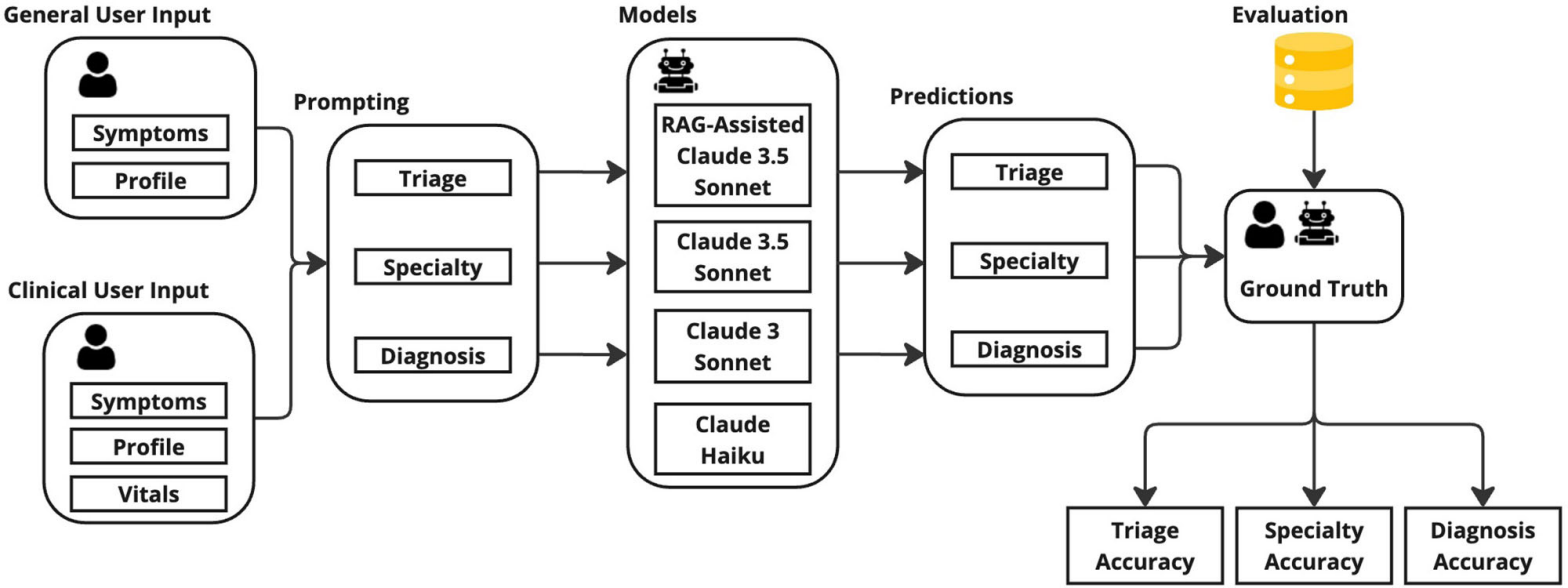
Workflow for clinical decision support using LLM. This workflow shows using LLMs in clinical decision support for referral, triage and diagnosis. The workflow begins with the input - general user input and clinical user input - followed by prompts engineered to predict triage, specialty and diagnosis. The predictions are generated using the Claude family models and a RAG-assisted Claude 3.5 Sonnet model. These predictions are then evaluated and compared with the ground truth to assess the performance of the LLMs.

In this study, we aimed to benchmark multiple LLM workflows on their ability to predict key aspects of clinical care: triage level in the form of the Emergency Severity Index (ESI)^19^, patient to medical specialty referral, and diagnosis based on symptoms (also referred to as history of present illness), patient information and initial vitals. The workflow is illustrated in Fig. 1. Using a dataset of 2000 real-world cases from the Medical Information Mart for Intensive Care (MIMIC-IV) database^20–22^, we evaluate the performance of several LLMs, specifically multiple versions of the Claude family^23,24^, as well as a retrieval-augmented generation (RAG) agentic workflow designed to mimic the clinical decision-making process.

This paper systematically evaluates the potential and limitations of these models in supporting clinicians with complex decision-making, showing promising results in their ability to assist effectively. With the increasing digitization of healthcare, the integration of AI-powered tools presents a promising opportunity to enhance clinical workflows and streamline patient-centered care. Such advancements will benefit both clinicians and patients.

## Results

### Curated MIMIC-ED dataset and model evaluation approach

**Fig. 2:**
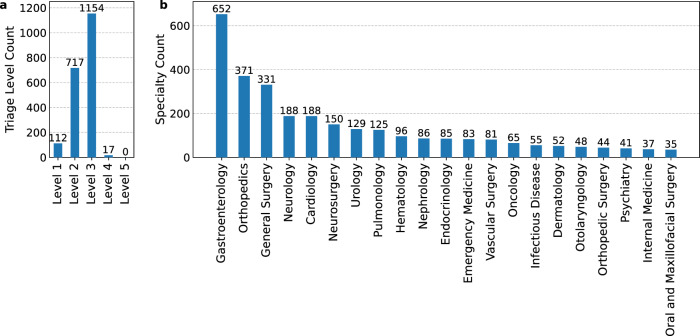
Triage levels and specialty distributions. **a** Shows the distribution of the triage level with the quantities assigned to each level and **b** distribution of medical specialties predicted from the diagnosis list, reflecting the specialties to which patient cases would be referred and consulted.

We created a curated dataset using the fully de-identified MIMIC-IV ED dataset^22,25^, consisting of electronic health records, together with the MIMIC-IV Notes^22,26^ to simulate clinical decision-making in an emergency department setting. Both datasets are modules from MIMIC-IV^20,22^. Details about the dataset and the preprocessing can be found in the Methods: Data Preprocessing. From the processed data, we extracted 2000 medical cases covering a wide range of medical conditions. Figure 2a displays the distribution of triage levels in the emergency department (ED), while Fig. 2b shows the specialties managing these cases, occurring more than 30 times. As expected in the ED, there were few triage level 4 and no triage level 5 cases (less severe), with most classified as triage level 3, followed by triage level 2, and a smaller number as triage level 1. This dataset has the advantage of not being directly publicly available, which makes it ideal for evaluating LLMs that otherwise tend to use publicly available test sets as part of their training data.

### Model selection and RAG-assisted LLM

We tested three models from the Claude family - Claude 3.5 Sonnet, Claude 3 Sonnet, and Claude 3 Haiku - due to their superior performance across multiple benchmarks, excelling in contextual understanding, efficiency, and handling specialized queries^23,24^ (see Methods: Model Selection for details).

Additionally to the stand-alone LLMs, we developed a retrieval-augmented generation (RAG) assisted LLM. Generally, a RAG method combines two components: a retriever system that extracts information from an external domain-specific knowledge source for the given query and an LLM that merges the retrieved context with the input query. The addition of extra information reduces hallucinations in the output and to allow for more precise and informed domain specific outputs^27^. In this study, the RAG-assisted LLM is used to enhance the performance of Claude 3.5 Sonnet, and utilizes a knowledge base of 30 million PubMed abstracts to improve its output with domain-specific context. A more in-depth explanation of the RAG workflow used in this paper can be found in the “Methods” section.

Due to privacy regulations surrounding the MIMIC-IV dataset, which prohibit its use with external application programming interfaces (APIs) like those provided by OpenAI (e.g., GPT-4), we utilized AWS Privatelink to privately connect to the Claude models supported by AWS services. More details are provided in the Methods: Model Selection. For each model we differentiated between two user types: general users, typically patients who provide only personal information and symptoms (referred to as the ‘history of present illness’ in the dataset), and clinicians in the ED, who can additionally retrieve initial clinical data, such as temperature, heart rate, respiratory rate, oxygen saturation, and blood pressure. While we recognize that making a definitive diagnosis requires further input, such as physical exams or laboratory tests, our approach seeks to replicate the decision-making process both for patients feeling ill at home and those arriving at the ED. This distinction allowed us to explore the capabilities of LLMs in both home settings, where users report symptoms, and ED settings, where preliminary clinical data is available.

### LLM performance in triage

In the context of emergency care, triage or acuity as it is mentioned in the MIMIC-IV-ED dataset refers to the severity of a patient’s condition and is commonly assessed using the Emergency Severity Index (ESI)^19^. This standardized triage tool classifies patients into five levels based on the urgency of their treatment needs, allowing healthcare providers to prioritize care more effectively. The levels range from ESI 1, which indicates patients requiring immediate life-saving interventions, to ESI 5, which represents cases where treatment can be safely delayed. The description for each level can be found in the Supplementary Table 1. This classification system plays a crucial role in emergency department operations, helping clinicians to allocate resources efficiently and address critical cases with minimal delay.

In our study, we assess the model’s capabilities to predict patient triage level for two user scenarios: a general user providing only symptom-based information and a clinician with additional access to initial clinical data. This evaluation aims to determine whether the models can be effectively integrated into the decision-making process as a first-pass aid to assist and help prioritize in triaging patients in the ED in real-time. The specific prompting details used for these cases can be found in the Methods: Prompts.

The results were assessed based once on exact match accuracy, where the predicted triage level matched the actual value, and a triage range accuracy, where predictions were considered correct if they were exactly or only one triage level higher than the actual level, except for the triage level 1, which has to be predicted as 1. The latter method compensates for the variability between different clinical judgements, arising from personal experience and knowledge, while only accepting triage level assignment if the LLM assigns a one level higher triage level than the ground truth. This approach reduces the risks of undertriaging while avoiding to overwhelm the system with exaggerated cautious overtriaged patients.

Models incorporating vital signs generally performed better in predicting the triage level than those using symptoms alone. RAG-Assisted LLM showed the highest exact match accuracy in both conditions. The addition of clinical data had a modest but positive effect on performance across all models and more recent models outperformed simpler ones.

**Fig. 3:**
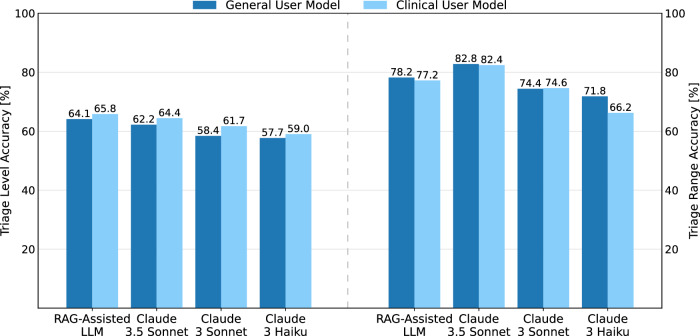
Triage level accuracy: exact match vs. range evaluation. This figure compares the model performance in predicting triage levels. The graph shows the accuracy [%] for two evaluation methods: exact match (left) and range evaluation (right). Results are displayed for both evaluation types, illustrating the differences in model accuracy across the different LLMs for triage level prediction.

**Fig. 4:**
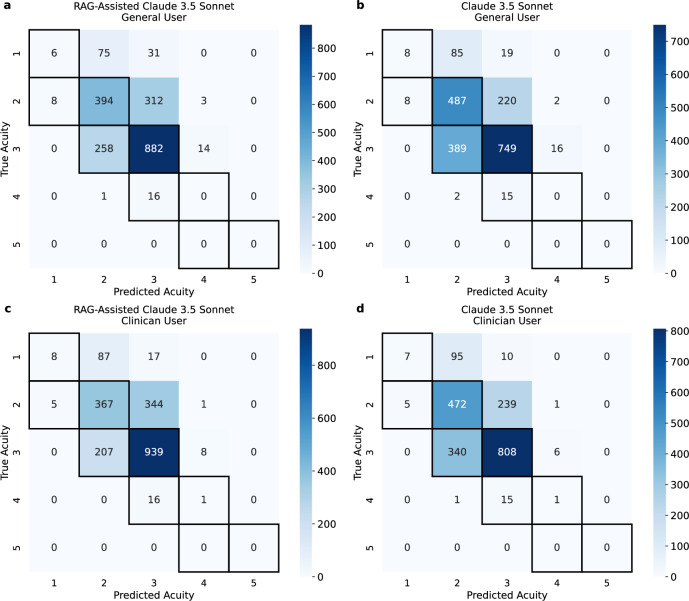
Confusion matrices for triage level prediction. This figure presents confusion matrices for the two best-performing models (RAG-Assisted Claude 3.5 Sonnet and Claude 3.5 Sonnet) in different user settings (General User and Clinical User) and evaluation methods (exact match and range evaluation). **a** and **b** show the confusion matrices for the general user setting and **c** and **d** show the confusion matrices for the clinical user setting. Diagonal values represent correct predictions in the exact match evaluation, while marked predictions indicate correct values for the triage range evaluation.

Under the triage range accuracy metric, Claude 3.5 Sonnet outperformed all other models. All the results are presented in Fig. 3 and Table 1. Figure 4 presents the confusion matrices for the two models that performed best in each accuracy evaluation. These matrices provide additional insight into the models’ behavior. It is important to note that while no model achieved high accuracy in predicting the most severe triage levels, none of the models confused the most critical cases with the least serious ones, and vice versa. This is a crucial finding, as it indicates that the models had difficulty accurately predicting cases at the extreme ends of triage severity, but they consistently recognized the difference between life-threatening cases and those of lower urgency.

**Table 1:** Model performance comparison across tasks and evaluation methods

User setting	Model	Triage level		Specialty		Diagnosis		Average
		Exact match	Range	Matched	At least one	Matched	At least one	
General User	RAG-Assisted LLM	**64.10**	78.20	77.12	86.35	69.43	80.85	76.01
	Claude 3.5 Sonnet	62.20	**82.80**	**78.26**	**88.05**	**70.22**	**82.00**	**77.26**
	Claude 3 Sonnet	58.35	74.40	78.10	87.70	70.17	81.55	75.05
	Claude 3 Haiku	57.70	71.80	77.86	87.10	67.39	79.60	73.58
Clinical User	RAG-Assisted LLM	**65.75**	77.15	77.28	86.45	69.77	81.70	76.35
	Claude 3.5 Sonnet	64.40	**82.40**	**78.86**	**88.55**	70.26	**82.10**	**77.76**
	Claude 3 Sonnet	61.65	74.55	77.72	87.15	**70.51**	82.05	75.61
	Claude 3 Haiku	59.00	66.15	78.02	87.05	67.46	79.30	72.83

Performance is presented as accuracy [%] on all tasks and with all evaluation methods. A bold value indicates the best-performing model and an underlined value indicates the second-best-performing model, determined separately within each user setting (general or clinical user) and within each evaluation method (exact match/matched or range/at least one) for each prediction task (triage level, specialty or diagnosis).

The improvement between the general user and clinical user models can be observed in Table 2, which shows a performance increase in triage level prediction across all models. This highlights how the LLM’s predictions improve when provided with more detailed information, similar to how a clinician makes more accurate decisions when given initial vitals. However, this improvement is not as apparent in the triage range evaluation. When the model misclassified the triage level, it is usually within the range of one level more severe. A slight decline in triage range accuracy was noted across most cases, except for Claude 3 Haiku, which struggled strongly to process the additional information from the initial vitals effectively.

**Table 2:** Clinical vs. general user settings

Model	Triage Level		Specialty		Diagnosis		Average
	Exact Match	Range	Matched	At Least One	Matched	At Least One	
RAG-Assisted LLM	1.65	–1.05	0.16	0.10	0.34	0.85	0.34
Claude 3.5 Sonnet	2.20	–0.40	0.60	0.50	0.04	0.10	0.51
Claude 3 Sonnet	3.30	0.15	-0.38	–0.55	0.34	0.50	0.56
Claude 3 Haiku	1.30	–5.65	0.16	–0.05	0.07	–0.30	–0.75

Performance improvement for each model from general user to clinical user setting.

### Predicting appropriate medical specialty referrals from patient data

We aimed to evaluate whether LLMs can assist in the specialty referral process. Accurate identification of the appropriate specialty for a patient is critical in ensuring they receive the most effective and timely treatment, which also reduces healthcare costs by minimizing unnecessary referrals. Since the MIMIC-IV-ED and MIMIC-IV-Notes datasets don’t contain exact information on the medical specialist the primary care doctors can consult or refer the patient to, we used Claude 3.5 Sonnet to create a ground truth by predicting the most likely specialist for each of the diagnoses of each patient. More details on this process and the used prompt can be found in Methods: Prompts. To validate this approach, we asked four clinicians to review a subset of the created ground truth to determine whether the assigned ground truth specialties appropriately matched the corresponding diagnoses. Additional information on the results and the acceptability of the LLM-generated ground truth among clinicians is provided in the subsection “Clinician Validation of LLM-Generated Specialties.” Further details about the clinician evaluation process can be found in the subsection “Reader Study” in the “Methods” section.

We evaluated the ability of LLMs to predict specialties in our two scenarios, the general user and clinical user models. For each scenario, we asked the model to predict the top three specialties that would handle the patient’s case based on the symptoms and the patient info, for the general user and adding the initial vitals for the clinical user. More insights on the two evaluation frameworks and the two user scenarios can be read upon in the Methods: Specialty evaluation Framework.

In the first evaluation, which checked each of the top three predicted specialties individually if it matches the specialties in the ground truth, Claude 3.5 Sonnet slightly outperformed the other models. However, the performance differences among all models were minimal, with all models showing similar accuracy across both general and clinical user scenarios.

**Fig. 5:**
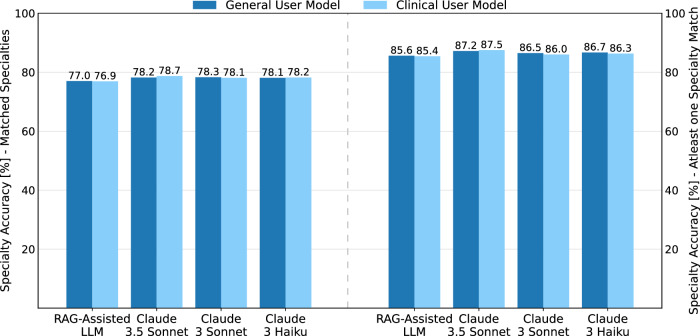
Specialty prediction accuracy: matched vs. at least one specialty match evaluation. This figure compares the model performance in predicting medical specialties. The graph shows the accuracy [%] for two evaluation methods: matched specialty (left) and atleast one specialty match (right). Results are displayed for both evaluation types, illustrating the differences in model accuracy across the different LLMs for specialty prediction tasks.

For the evaluation that focused on checking if at least one of the top three predicted specialties is predicted correctly, Claude 3.5 Sonnet had the highest performance, while overall performance differences remained small across all models. The results are illustrated in Fig. 5 and Table 1.

The improvement shown in Table 2 between the general user and clinician user models was most evident in the Claude 3.5 Sonnet model, with only minimal improvements seen in the RAG-assisted LLM and Claude 3 Haiku. In contrast, Claude 3 Sonnet experienced a negative impact when provided with additional information about the initial vitals. Predicting the appropriate specialty relies on several factors. Symptoms need to be clear and accurate, but it’s common for symptoms to fall under the expertise of multiple specialists, and often additional tests are required to narrow down the appropriate referral. In this study, the specialty was defined by the patient’s discharge primary diagnosis, meaning the diagnosis was made after several tests and possibly after days of observation. As a result, the addition of initial vitals may not significantly influence specialty prediction, as more detailed information becomes available only later in the patient’s care.

The evaluation of specialty frequencies, which can be found in the Supplementary Fig. 7 shows that in the best model, clinical user Claude 3.5 Sonnet, general surgery, emergency medicine, infectious diseases, and internal medicine are overrepresented, while the underrepresentation of orthopedics is nearly balanced by the higher occurrence of orthopedic surgery. The same tendencies can be seen in the other models.

The performance of LLMs in predicting the specialties shows that LLMs are generally well-suited to assist in medical referrals by offering a variety of relevant specialty options.

### Evaluating LLM workflows for diagnostic accuracy

In the process of clinical decision-making, we evaluated whether LLMs can assist in predicting the diagnosis or diagnoses a patient might have. We conducted this evaluation in our two settings like described before, the general user and clinical user setting. More on the evaluation framework can be found in the Methods: Diagnosis Evaluation Framework. To compare the predicted diagnoses to the ground truth diagnoses, we used Claude 3.5 Sonnet as a judge. Additionally, to validate this approach of using an LLM as an evaluator, we asked four clinicians to review a subset of the data and compare the predicted diagnoses to the ground truth diagnoses. A detailed explanation of this process can be found in the subsection “Reader Study” in the “Methods” section, and the results are presented in the subsection “Inter-Rater Agreement on Diagnosis Evaluation” in the “Results” section.

**Fig. 6:**
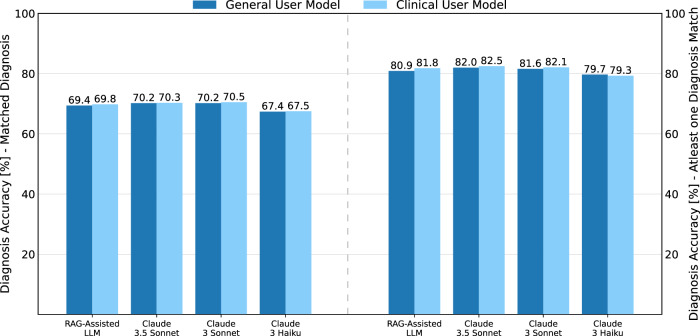
Diagnosis prediction accuracy: matched vs. at least one diagnosis match evaluation. This figure compares the model performance in predicting medical diagnoses. The graph shows the accuracy [%] for two evaluation methods: matched diagnosis (left) and atleast one diagnosis match (right). Results are displayed for both evaluation types, illustrating the differences in model accuracy across the different LLMs for diagnosis prediction tasks.

In our evaluation of LLMs’ ability to assist in predicting patient diagnoses, we found small differences in performance between models. In the first evaluation, in which each diagnosis was compared to the ground truth, Claude 3.5 Sonnet and Claude 3 Sonnet performed equally well for the general and clinical user setting. In the second evaluation, where the goal was to predict at least one correct diagnosis for each patient, all models demonstrated stronger performance. All results are presented in Fig. 6 and in Table 1.

Improvements in the clinical user model over the general user model is particularly notable for the RAG-assisted LLM, as shown in Table 2. This suggests that the knowledge provided to the LLM during the RAG workflow has enhanced its diagnostic skills, particularly in interpreting and utilizing current initial vitals. Predicting or defining a diagnosis, like specialty referral, requires a significant amount of information, much of which is difficult to gather upon a patient’s arrival to the ED. This complexity underscores the challenges of early diagnosis in such fast-paced settings, where many crucial details are still emerging.

### Intra-model agreement

The agreement between the models can be seen as a measure of quality, as high agreement indicates that similar patterns and trends are captured in the responses, indicating robustness and reliability of the predictions. The analysis of inter-model agreement for the diagnosis data was omitted as this data has the highest variability and therefore requires assessment by an LLM judge.

Comparisons of inter-model agreement across the triage and specialty datasets are shown in Table 3, with full results available in Supplementary Table 2. The intra-model agreement analysis showed the highest consistency between the general user model and the clinical model for all models. This suggests that the different inputs to the same model do not significantly alter or improve responses, but also that the models - particularly Claude 3.5 Sonnet and RAG-assisted LLM - show consistent performance across different user settings.

**Table 3:** Average inter-model agreement of the triage level and specialty predictions

Category	RAG-Assisted LLM	Claude 3.5 Sonnet	Claude 3 Sonnet	Claude 3 Haiku	Average
Between General and Clinical User	85.94	89.74	84.95	83.54	86.04
To RAG-Assisted LLM	-	83.88	76.64	74.34	78.29
To Claude 3.5 Sonnet	83.88	-	77.19	73.35	78.14
To Claude 3 Sonnet	76.64	77.19	-	75.65	76.49
To Claude 3 Haiku	74.34	73.35	75.65	-	74.44

Average inter-model agreement [%] for different categories over triage level and specialty. The “Between general user and clinical user” category shows the average agreement between the corresponding general user model and clinical user model, while the other categories show the average agreement to a certain model of the same type (general user to general user and clinical user to clinical user). Agreement to the same model is omitted to avoid distorting the average.

RAG-Assisted LLM demonstrated the highest average agreement across all models, closely followed by Claude 3.5 Sonnet, while the highest single inter-model agreement was between Claude 3.5 Sonnet and RAG-assisted LLM.

The high agreement between the models underlines their consistency in many cases, but the variation in agreement suggests that different models correctly classify different cases. This indicates that if we could determine which model excels at specific classifications, we could potentially reduce the overall error rate by a significant margin.

### Clinician validation of LLM-generated specialties

To evaluate the accuracy and reliability of the LLM-generated specialties, we asked four clinicians to independently review a subset of the dataset. Each clinician assessed the LLM-generated specialty and its appropriateness for a given diagnosis. They categorized each specialty as either Correct, Partially Correct, Reasonable but Suboptimal or Incorrect. Two clinicians reviewed the same subset to provide an independent evaluation and objectivity. A more detailed description can be read upon in the Methods “Reader Study”.

The results presented in Table 4 show the clinicians’ positive assessment of the LLM’s performance in creating the specialty ground truth. On average across all clinicians, a significant 81.5% of the predictions were rated as Correct, which shows the LLM’s promising ability to align closely with clinical expectations. Partially Correct and Reasonable but Suboptimal predictions are considered as acceptable choices. When the accurate and acceptable categories are combined, the acceptability rises to 97.03%, showing that almost all recommendations are clinically relevant. Notably, the overall error rate was exceptionally low at just 2.63%. However, a closer look reveals that Clinician 2, with a higher error rate of 9.15% compared to the average error rate of 0.68% for the other clinicians, rated predictions more stringently. This highlights the variations in human evaluations, which is likely influenced by differences in experience and individual judgment. These differences can also be observed when comparing the two confusion matrices of the two clinician pairs, provided in the Supplementary Fig. 8 and 9.

**Table 4:** Clinician validation of LLM-generated specialties: accuracy, acceptability, and error rates

Clinician	Accurate [%]	Acceptable [%]	Accurate & Acceptable [%]	Error Rate [%]
Clinician 1	93.77	6.23	100	0
Clinician 2	82.05	8.79	90.84	9.15
Clinician 3	81.91	17.06	98.98	1.02
Clinician 4	68.94	30.34	98.98	1.02
Average	81.5	15.53	97.03	2.63

Average Accuracy [%], Acceptability [%], Combined Accuracy and Acceptability [%], and Error Rate [%] for the ground truth specialties generated by the LLM, as evaluated by clinicians. The results are shown for each clinician and the overall average across all clinicians.

These results suggest that LLMs perform well in generating a ground truth specialty, aligning closely with clinical expectations with high acceptability. The low error rate highlights their reliability, though the variations in clinician evaluations reflect differences in judgment among clinicians. LLMs have strong potentials for specialty recommendations, while human oversight remains important for complex cases.

### Inter-rater-agreement on diagnosis evaluation

To trust the LLMs predictions in clinical decision-making, it is essential to evaluate how well their outputs align with human evaluation. Therefore, we aimed to assess the inter-rater agreement between the two LLMs, Claude 3.5 Sonnet and the RAG-assisted LLM, and clinicians in comparing predicted diagnoses to ground truth diagnoses. Four clinicians participated in the evaluation, where each pair of clinicians assessed the same subset to ensure independent evaluations and to compare their assessments. They categorized the LLM-predicted diagnoses into four levels: Exact Match, Clinically Equivalent, Clinically Related, or Incorrect. The LLMs provided a binary evaluation, deciding whether the predicted diagnosis exactly matched the real diagnosis or fell within a broader category related to the real diagnosis. More details about the reader study and the LLM as a judge can be found in “Methods”.

To measure alignment, we mapped the LLMs’ binary outputs to the clinician ratings. True was matched with Exact Match or Clinically Equivalent, while False was aligned with Clinically Related or Incorrect. Each subset was reviewed by two clinicians, allowing us to evaluate agreement under two conditions: Union and Intersection. The results are presented in Table 5.

**Table 5:** Clinician validation of llm-generated diagnoses: inter-rater agreement

Clinician	Inter-Rater Accuracy [%]		Inter-Rater Union Accuracy [%]		Inter-rater intersection accuracy [%]	
	Claude 3.5 sonnet	RAG-assisted LLM	Claude 3.5 sonnet	RAG-assisted LLM	Claude 3.5 Sonnet	RAG-assisted LLM
Clinician 1	90.43	90.11	95.22	95.37	75.44	73.84
Clinician 2	80.22	79.11				
Clinician 3	76.51	77.78	96.03	94.44	66.03	65.87
Clinician 4	85.56	82.52				
Average	83.18	82.38	95.62	94.91	70.74	69.86

Average Inter-Rater-Agreement Accuracy [%] for Claude 3.5 Sonnet and RAG-assisted LLM compared to clinician evaluations. Inter-Rater Accuracy is reported for each clinician, along with combined values for the Union and Intersection evaluations. For the Inter-Rater Union and Intersection, a single value is reported for each pair of clinicians: one value for the Union and one value for the Intersection of Clinicians 1 and 2, and similarly for Clinicians 3 and 4.

The evaluation of LLM evaluating predicted diagnoses shows a strong alignment with human evaluation, particularly in collaborative clinical scenarios, referred here as the union scenario. The union scenario means an LLM evaluation was considered correct if at least one of the two clinicians agreed with its evaluation (e.g., marking a “True” as Exact Match or Clinically Equivalent). This approach highlights the collaborative nature of clinical practice, where different perspectives are required to enhance decision-making and arrive at a more comprehensive understanding. Under this scenario, Claude 3.5 Sonnet achieved a high accuracy of 95.62%, while the RAG-assisted LLM followed closely with 94.91%. These results emphasize the models’ ability to align with the reasoning of at least one human expert in most cases.

The Intersection scenario represents stricter conditions, where an agreement among clinicians is required, such as during critical multidisciplinary team discussions. In this scenario, an LLM evaluation was considered correct only if both clinicians agreed with its evaluation (e.g., both rated a “True” as Exact Match or Clinically Equivalent). The results here show lower but still significant accuracy, with Claude 3.5 Sonnet achieving 70.74% and the RAG-assisted LLM reaching 69.86%. This outcome shows the challenges of getting full agreement among human experts.

These results demonstrate that LLMs perform well in collaborative and flexible clinical scenarios, as shown by the high accuracy in the union condition. However, the stricter Intersection results reveal the challenges of achieving full agreement, even among human experts, due to differences in perspectives and levels of experience. This variation can also be seen in the individual inter-rater accuracy between clinicians and the LLMs, which ranged from 76% to 90% for both Claude 3.5 Sonnet and the RAG-assisted LLM. These differences highlight the variability in how clinicians assess LLM-evaluated answers and interpret predictions. These differences can also be observed in the evaluation results between the two clinician pairs, as shown in the confusion matrices provided in the Supplementary Fig. 10 and 11. While one pair showed higher agreement in the “Exact Match” category, the other pair demonstrated a more distributed matching across different criteria. These findings suggest that while LLMs align well with human evaluation, especially in collaborative settings, there is still room for improvement in achieving full consensus. The results also highlight the potential value of using LLMs as complementary tools in multidisciplinary discussions, where diverse perspectives can enhance decision-making processes.

## Discussion

Advances in large language models (LLMs) are beginning to reshape how clinicians approach medical decision-making. These models have already proven useful in more structured tasks, like medical licensing exams, but how they can be used in real-world patient care is still being studied. We explored the potential of LLMs with and without RAG assistance, to support clinical decision-making by benchmarking their performance on 2000 real-world medical cases from the MIMIC-IV-ED dataset. We wanted to assess their ability to predict diagnoses, recommend specialists, and determine the urgency of care. Our results highlight both the promise and limitations of LLMs in the clinical decision process, offering insights into their potential role in healthcare.

Our results suggest that LLMs and the RAG-assisted LLM can support clinical decision-making, but their effectiveness varies depending on the task. Claude 3.5 Sonnet generally performed slightly better across most tasks, but the RAG-assisted LLM offered an important advantage: the ability to use external, trusted references. This feature helps reduce the risk of hallucinations^28–31^ and adds a layer of fact-checking^32,33^, which is crucial in clinical settings where accuracy is crucial. The RAG-assisted LLM, compared to its base model Claude 3.5 Sonnet, showed a different pattern of improvement when using the clinical user setting (with additional patient vitals data), as demonstrated in Table 2. The RAG-assisted LLM benefited significantly from the extra vital information in the triage level and diagnosis tasks, though less so in the specialty task. In contrast, Claude 3.5 Sonnet showed improvements in the triage level and specialty tasks but gained less from the vital signs in the diagnosis task.

The RAG workflow allows the model to incorporate external sources from a research context, helping to provide a more informed perspective on the input. We hypothesize that the available external information likely emphasizes the relationship between vital signs and triage level or diagnosis, but not as much between vital signs and the corresponding referral specialty. Therefore, with the background knowledge provided by the RAG workflow, it makes sense that this model benefits more from additional vital signs in the domains of triage level and diagnosis. This also suggests that the RAG workflow improves the model’s performance in cases where current research findings are particularly relevant.

This is further highlighted by the RAG-assisted LLM’s strong performance in terms of exact accuracy on the triage level data with vital signs information, which is likely to be well-represented in available research resources. However, this does not necessarily help the model with range accuracy, as research sources are unlikely to guide the model in predicting more severe over less severe.

However, the benefit of incorporating more clinical information was not seen in simpler models like Claude 3 Haiku, and only minimal gains were observed for Claude 3 Sonnet when predicting specialties. This is in line with previous findings that LLMs struggle with nuanced clinical data, like interpreting abnormal lab results or subtle symptoms^8^. It also explains why none of the models achieved high accuracy in predicting the most severe triage patients, as these models are not equipped to follow numeric-based guidelines effectively. More advanced models, like Claude 3.5, showed they are better at handling these complexities.

Accuracy in the medical domain remains a significant challenge for LLMs, particularly in predicting triage levels, as all models showed both over-triaging and under-triaging. Assigning a triage level in a real-world setting demands a high degree of clinical judgement and careful consideration of the patient’s conditions and resources needed. For the triage prediction, we acknowledge that using clinical notes may introduce bias since triage determination is typically made prior to the patient being fully assessed by a physician.

To limit the bias, we extracted the HPI, through our data processing, attempting to include only the reason for a patient’s admission and first impression by a clinician as the earliest recording of the patient’s symptoms documented in the physician’s notes. Ideally, one would use real-time audio or video recordings of the initial patient contact with medical personnel—such data is unfortunately not available. While this study is purely an academic evaluation of various LLMs’ performance, the difficulty LLMs demonstrate in assigning accurate severity levels - in particular at the extreme ends - highlights their current limitations in independently handling these tasks. Nonetheless, the performance on this task remains a meaningful indicator of the overall quality of LLMs, their potential as an assisting tool and their promise for future clinical applications.

Future work could focus on improving LLMs’ numerical handling of laboratory values to enhance their ability to interpret clinical data accurately. Additionally, a deeper examination of how clinicians make decisions when defining ESI levels is essential. This could provide valuable insights to improve LLMs to better replicate clinician performance in real-world scenarios.

A critical aspect of utilizing LLMs in clinical decision-making is the importance of prompt design. In our study, we experimented with various prompts to guide the models effectively, and it became evident that how a task is framed significantly impacts the quality of the results^34,35^. While we observed promising outcomes, it is clear that a more focused approach to prompt engineering would be highly beneficial^36–40^, particularly when combined with the context of external sources provided by the RAG workflow. One interesting observation was the differences in performance between the LLM models. The models did not always agree on their predictions, which points to both a limitation and an opportunity. The results on intra-model agreement reveal that the models do not completely overlap in their predictions, suggesting that they might function as a “mixture of experts” when combined. Leveraging this diversity in predictions could lead to improved outcomes by utilizing the strengths of each model in different contexts. Additionally, higher agreement between models can be seen as a measure of quality, as it indicates that similar patterns and trends are being captured, contributing to the robustness and reliability of the predictions.

A similar pattern of variability and complementarity observed in the intra-model agreement was also reflected in our evaluations involving clinician reviews. It highlights how differences in perspectives and expertise can influence decision-making. In the review of LLM-generated specialties, clinicians showed variation in their assessments, shaped by individual judgment and experience. Similarly, in the evaluation of LLM-predicted diagnoses, the union condition demonstrated strong alignment with the LLM-judge, emphasizing the collaborative nature of clinical environments where different viewpoints can complement each other. However, the stricter intersection condition revealed the challenges of achieving full agreement among clinicians. This shows the complexity of consensus in medical decision-making.

Finally, while our study establishes benchmark tasks and resources for clinical decision-making, the next step will involve refining the RAG-based model and similar approaches, and focusing on integrating them more effectively into clinician workflows. Beyond helping healthcare providers, these models can also benefit patients directly. For those experiencing symptoms at home, LLMs can provide an initial assessment, giving patients an indication for the severity of their condition and recommending which specialist to visit. This empowers patients to make more informed decisions about their care.

We do not propose a direct clinical deployment, but it is still relevant to mention that any clinical deployment would need to address significant regulatory concerns regarding AI in clinical tasks, especially those involving LLMs for referral, triage, and diagnosis. These specific tasks fall under “determining eligibility for healthcare” (5a) and “emergency healthcare patient triage systems” (5 d) and are consequently classified as high-risk in Annex III of the EU Artificial Intelligence Act (AIA)^41^. As such, clinical implementation must comply with the AIA’s requirements for robust validation (Art. 57), potentially including multiple external validation sets and assessments of performance on edge cases. Moreover, the Act mandates continuous post-market monitoring (Art. 72) and reporting of serious incidents (Art. 73), highlighting the ongoing nature of AI system validation beyond initial approval.

Nevertheless, it is impossible to rule out “leaky deployment” of LLM models, where physicians would start using openly available models as helper systems in their clinical routine. Therefore, open and strict benchmarking of LLM performance on various sets of clinical tasks is of utmost importance for both the medical community and general populace.

While our study focused on Claude models, it is relevant to consider how other advanced LLMs might perform in similar clinical decision-making contexts. Newer reasoning-focused models, such as the OpenAI models with their “Deep Research” function or Claude 3.7 Sonnet^42^ with its “extended thinking mode”, could offer substantial advantages in handling complex medical cases. The rapid development of these models highlights the need for objective benchmarks and continuous evaluation to ensure their reliability, and usefulness in clinical applications.

The results presented here are primarily of academic interest, providing a first highly needed benchmarking of LLMs in the AI-assisted clinical-decision-making process, as we believe these systems require further refinement and validation before any potential clinical deployment. Our study shows that LLMs cannot replace clinicians in independently performing complex medical decision-making. However, they demonstrate potential as supportive tools, assisting clinicians by providing relevant insights and information.

This highlights how LLMs are more suitable for alternative use cases, such as educational resources for inexperienced clinicians, supplementary resources for patients, or background safety screenings. Ultimately, their effective integration into healthcare will rely on thorough testing, ongoing improvements, and well-defined roles within clinical workflows.

## Methods

### Data preprocessing

Our goal was to develop a model capable of predicting the specialty, triage level, and diagnosis for patients in an emergency department (ED) setting or those experiencing symptoms at home. Since we aimed to evaluate the difference in model performance based on whether the information was entered by the patient themselves or a clinician, we designed our dataset accordingly. For the general user, we required two main inputs: a description of the patient’s symptoms and some basic patient information. For the clinical user we added the initial vitals signs, such as temperature, heart rate, respiratory rate, oxygen saturation, and blood pressure, which can be measured upon arrival at the ED.

We processed and created our curated dataset using the MIMIC-IV ED dataset^22,25^ in conjunction with the MIMIC-IV Notes^22,26^ dataset, both modules from MIMIC-IV^20–22^, to support clinical decision-making in an emergency department setting. The MIMIC-IV ED dataset contains extensive information from patients admitted to the emergency department, while the Notes module provides valuable unstructured clinical notes of these patients.

**Fig. 7:**
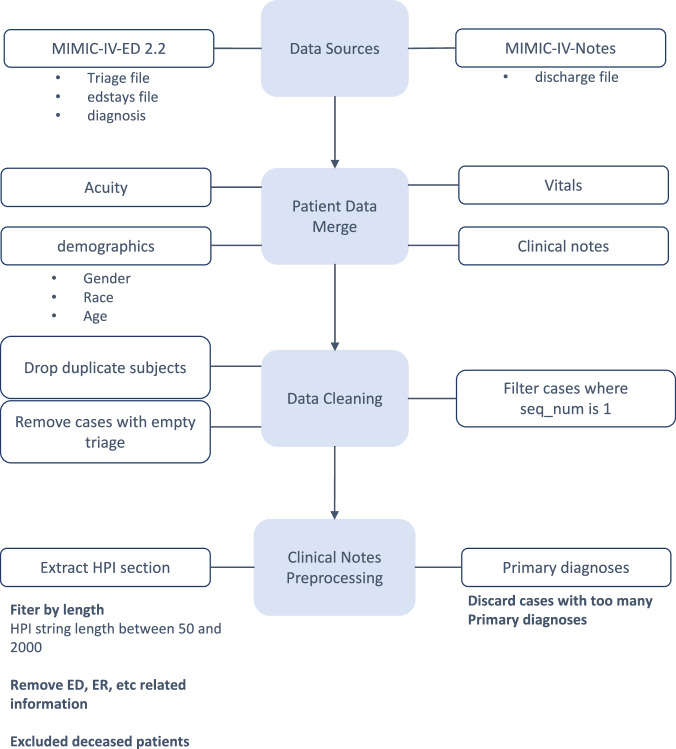
Flow diagram for the data preprocessing. This figure illustrates our data preprocessing pipeline. From Physionet we utilized MIMIC-IV-ED 2.2 and MIMIC-IV-Note. The necessary data tables from each data source were merged. Next, the merged data undergoes processing and cleaning. Finally, we process the clinical notes to extract the relevant information - history of present illness and primary diagnoses.

The data processing pipeline is presented in Fig. 7. First, we merged the necessary data tables from each source. Triage information was obtained from the MIMIC-IV-ED “triage” file, while the patient demographics such as race, and gender were extracted from the “edstays” file. The age specifically was extracted from the MIMIC-IV “patients” file. The initial vital signs were extracted from the MIMIC-IV-ED “triage” file, and the unstructured clinical notes were extracted from the MIMIC-IV-Note “discharge” file.

Initially, we extracted relevant discharge notes from MIMIC-IV-Note dataset and linked them with the patient records from the MIMIC-IV-ED 2.2 “edstays” file using the *stay_id*. We then merged the triage information and the patient demographics - gender, race and age) from the respective files, and integrated the initial vital signs. During this merging process, we dropped duplicate subject entries, removed cases with missing triage information, and filtered the records to retain only those with sequence number (seq_num) equal to 1. This ensures the uniqueness of the ED stays. We also excluded patients that had died.

A separate preprocessing step was applied for the unstructured clinical notes. Specifically, we selected only the patients that had a history of present illness in the unstructured notes. We extracted the history of present illness paragraph from the discharge notes - discarding any other information included in the notes. We further selected only cases with HPIs that had a string length between 50 and 2000 characters, to avoid getting too short or too long HPIs. We additionally removed any entries that mentioned “ED” or “ER”, as these references did not include any necessary information regarding the patient’s symptoms or how the patient was feeling.

Additionally to extracting the HPI, we extracted the diagnoses list for each patient from the clinical notes. These lists were typically divided into primary and secondary diagnoses. For our evaluation, we used only the primary diagnoses and discarded cases that had more than 15 primary diagnoses, as most cases had up to 3 diagnoses. This approach ensures that the dataset accurately reflects patient information and vital signs at the time of emergency department triage, offering a comprehensive view of early-stage clinical decision-making.

### Prompts

We created a series of prompts to guide the LLM in performing specific clinical tasks. These included predicting the triage level, predicting the specialty and diagnosis both together as they are both related and complement each other. Additionally, we used the prompt creating a ground truth referral specialist, and using the LLM as a judge to compare predicted diagnoses with the true diagnoses. To decide on these prompts, we experimented with several variations of the prompts on a subset of data that was not included in our evaluation to refine the prompts to our tasks. To ensure consistent and reliable outputs, we set the temperature parameter to zero during these experiments. We observed that the results were identical across runs, with no variations. Based on this observation, and given the cost constraints of running the LLM multiple times, we decided to run the predictions only once for the final evaluation. Additionally, our goal is to evaluate the LLM’s performance in a scenario that mimics a clinical environment, where a clinician would typically rely on the LLM’s first output rather than running it multiple times. By focusing on the first output, we aimed to test the reliability and practical usability of the LLM in such a setting.

Each prompt begins by setting the system’s role, such as, “You are an experienced healthcare professional with expertise in medical and clinical domains”, followed by clear task instructions. We also provided the data necessary for each task and specified how the LLM should format its responses, ensuring concise answers within predefined tags. The different prompts can be seen in the Supplementary Fig. 1-6.

### Model selection

To comply with privacy regulations restricting the use of the MIMIC-IV dataset with external APIs like OpenAI’s GPT-4o and the Claude family models, we employed AWS Privatelink to securely connect to the Claude models hosted on AWS. This kind of evaluation reduces the likelihood that the data has been previously seen by the LLM models, which cannot be guaranteed when using publicly available datasets.

Claude 3.5 Sonnet, Claude 3 Sonnet, and Claude 3 Haiku are advanced LLMs developed to enhance natural language understanding, with improvements in performance and efficiency across multiple benchmarks over their predecessors, including GPT-4o, GPT-4T, Gemini 1.5 Pro and Llama 3 400B^23^. They excel in contextual understanding, efficiency, and their ability to handle specialized queries. This makes them well-suited for applications in clinical decision-making, where precision and adaptability are essential.

Claude 3 Haiku is the fastest and most compact model in Anthropic’s Claude 3 family. It excels in tasks where it requires quick analysis and response times^24^, making this feature suitable for the clinical-decision process.

Claude 3 Sonnet is a balanced combination of speed and intelligence, offering significant improvement in reasoning and accuracy. This model is versatile, handling complex text generation, analysis and reasoning^24^.

Claude 3.5 Sonnet is built on the foundations of Claude 3 Sonnet with further enhancement in speed and intelligence. It excels in different tasks like reasoning and question answering, while being faster and cost-efficient relative to the previous models. It has shown competitive or superior performance in a variety of language-based tasks^23^.

### RAG-assisted LLM

**Fig. 8:**
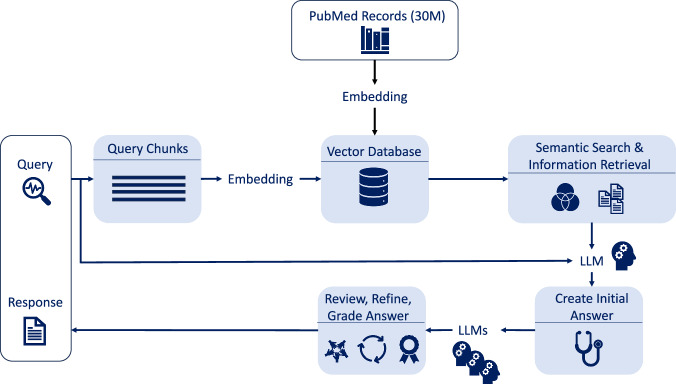
Workflow of the RAG-assisted LLM. The workflow starts with query decomposition, breaking down patient queries into smaller chunks. These chunks are embedded and undergo a semantic similarity comparison with the embeddings of 30 million PubMed abstracts to extract the most relevant information. The retrieved information is then combined with the query, and the LLM generates responses supported by the source references. An iterative critique-refinement loop further enhances the outputs by identifying gaps, refining responses, and ensuring alignment with the query.

A RAG-assisted LLM approach involves two components: a retrieval mechanism that gathers the relevant information corresponding to the query from a specific external knowledge base, and a language model that integrates the retrieved information with the query to produce a response that is both grounded in the external knowledge base and tailored to the specifics of the given query. This method has shown improvements in both accuracy and reliability, which significantly reduces false or misleading information, referred to as hallucination, and produces more factual, context-aware outputs^28–31^. In this study, the framework is implemented using Claude 3.5 Sonnet as the LLM component and incorporates a multi-step process where the LLM plays a key role in refining and enhancing query processing and answer generation. The workflow is represented in Fig. 8.

The workflow starts with a query decomposition, breaking down the patient’s query into smaller queries. This process allows RAG systems to break down the input into its smaller key components and retrieve the most relevant information for each component. This idea is supposed to mimic the natural way humans approach understanding by breaking down complex information into smaller parts to focus on each element individually.

The knowledge base supporting this workflow consists of 30 million PubMed abstracts, which have been converted into embedding vectors and stored as a knowledge high-dimensional vector database. This allows the system to measure semantic similarity by comparing these vectors to those in the knowledge vector database. By identifying the closest matches, the system retrieves the most relevant information for the given query.

The LLM uses the retrieved information alongside the query to generate a response that is supported by the retrieved data, while also providing the source PubMed references for further review. An additional layer in the workflow tries to enhance the performance through iterative loops of critique, refinement, and retrieval. In these loops, the LLM evaluates the generated responses, identifies gaps or inaccuracies, and refines the output as needed. We used an LLM to evaluate the output and determine whether it was sufficient for the given query. This iterative process intends to achieve higher accuracy alignment with the query, to create more precise and reliable outputs.

### Triage level evaluation framework

The triage level is based on the Emergency Severity Index (ESI)^19^, which consists of five levels, as outlined in the Supplementary Table 1. We evaluate the model’s triage level predictions using two different assessment frameworks. The first is a straightforward comparison between the predicted triage level and the ground truth, with accuracy as the metric. The second evaluation framework uses a triage range approach, accounting for the variability in clinical judgment when assigning triage levels. The ESI is typically determined by a clinician assigning a score based on their assessment of a patient’s condition. Although there are defined levels within the ESI system, ranging from 1 to 5, the assignment of these levels can vary due to the clinician’s intuition and experience. In some cases, clinicians may lean on the side of caution, assigning a more severe level to avoid the risk of patient deterioration or the possibility of misclassifying a patient as less critical than they actually are. To account for this variability, our evaluation allows some flexibility in model predictions. If the real triage level value is 1, the model must predict 1, as immediate life-saving intervention is required. For a real value of 2, the model can predict either 1 or 2, ensuring patients needing urgent care aren’t harmed by overclassification. Similarly, if the real value is 3, the model can predict 2 or 3, and so on—up to a real value of 5, where the model can predict either 4 or 5.

### Specialty evaluation framework

To assess the performance of LLMs in recommending appropriate medical specialties, we developed two distinct evaluation scenarios: one tailored for the general users and another for clinical users. In each scenario, the models generated the top three specialty recommendations based on the available patient information. For the general user case, this input consisted of a description of the symptoms and basic patient information, while for the clinical user case, the input was augmented with the patient’s initial vital signs. For the general user setting, we implemented this evaluation with the German healthcare system in mind, where patients can choose any specialist without prior consultation with primary care or emergency care specialists. For the clinical user, we designed the evaluation to assist primary care doctors in referring patients to a specialist or seeking consultation as needed.

Since the MIMIC-IV-ED and MIMIC-IV-Notes do not include information on whether a consultation is necessary - and we could not compensate for this missing detail - we put our focus on evaluating the question “which specialist would be most helpful given the symptoms at hand.” As the datasets lack exact information on the medical specialist each patient visited, we used Claude 3.5 Sonnet to predict the most likely specialist for each diagnosis for each case, given that patients often present with multiple diagnoses rather than just one, thereby establishing the ground truth for this study.

Predicting a single specialist would be insufficient and unfair to the model when comparing its performance to the ground truth consisting of several specialties. In fact, it’s not uncommon for a patient to suffer from several medical conditions simultaneously, each requiring attention. To address this complexity, we chose to predict the top three specialists for each case. An Example is provided in Table 6.

**Table 6:** Example diagnoses, ground truth and predicted specialty

Primary Diagnoses	Ground Truth Specialties (Claude 3.5 Sonnet)	Predicted Specialties (Claude 3.5 Sonnet)
• Alcohol withdrawal • Pancreatitis • Thrombocytopenia • Schizoaffective • HIV	• Addiction Medicine • Gastroenterology • Hematology • Psychiatry • Infectious Disease	• Gastroenterology • Hepatology • Addiction Medicine

Example of a case with primary diagnoses, their corresponding created ground truth, and the predicted specialties for the case

This approach provides a more realistic comparison and offers clinicians and patients multiple possibilities to consider, reducing the risk of bias toward a single diagnosis. Ultimately, the LLM serves as a support tool, providing valuable insights, while the clinician makes the final, informed decision based on both the LLM’s recommendations and their own expertise.

### Diagnosis evaluation framework

As mentioned in the specialty evaluation previously, patients often come in with more than one diagnosis. To reflect this, we predicted a top three list of diagnoses for each case. We then compared each of these predictions to the actual diagnoses. Notably, we examined the time from admission to release and confirmed that all cases used in our evaluation had a stay duration of less than one day. This minimizes the possibility to include diagnoses that might arise from later during hospitalization.

To make the comparison more accurate, we used an LLM judge to decide if the predicted diagnosis either matched the ground truth or fit into a broader category of one of the actual diagnoses. This way, we accounted for differences in wording while still ensuring a fair evaluation. Additionally, on a subset of the dataset, we involved four clinicians who compared the predicted diagnoses to the ground truth diagnoses and reviewed them. More details about this process can be found in the subsection “Reader Study”.

We employed two evaluation methods for assessing the model’s performance in predicting the correct specialty. The first method evaluated whether each predicted specialty appeared in the ground truth list. For each patient, we counted how many specialties were correctly predicted and then divided that number by the length of the shorter list, either the ground truth or the prediction list.

For example, if the ground truth for a patient included only one entry, a cardiologist, and the model predicted three specialists—one cardiologist, one general medicine, and one electrophysiologist—only the cardiologist would be considered correct. Although general medicine and electrophysiology could also be relevant in some cases, our evaluation was specifically set to match the ground truth. This ties into a point discussed in the paper, where we explore how a single diagnosis might be managed by multiple specialists, a factor we plan to address in future work.

In this example, since only the cardiologist was correctly predicted, the patient would receive one point, which is then divided by the length of the shorter list (in this case, one, as the ground truth had only one entry). So, the score for this patient would be 1. If the ground truth had included two specialties, and the model only correctly predicted one out of three, the score would be 0.5. The total points across all patients were then summed and divided by the total number of patients to calculate the overall accuracy.

The second evaluation framework was simpler, focusing on whether at least one of the predicted specialties appeared in the ground truth list. If any one of the model’s predicted specialties matched one of the ground truth specialties, the prediction for that patient was considered successful.

### LLM judge

For our study, we utilized LLMs to evaluate and compare the accuracy of predicted diagnoses for a given set of patient cases. This evaluation aimed to assess the model’s diagnostic capabilities by comparing the predicted diagnoses with those listed in the patient’s medical records. The prompt for the evaluation can be found in the Methods: Prompts.

The model was given the true list of diagnoses for each patient, along with three predicted diagnoses.

It was then asked to determine if the predicted diagnosis matched any of the primary diagnoses by focusing on semantic equivalence and meaning, or if it fell under a broader category related to the real diagnosis. Since LLMs may use different phrasing for the same concept, which string-matching algorithms could miss, the model was asked to evaluate whether the predicted diagnosis matched the real one or was related to it in a broader sense. If it did, the model returned “True”, ensuring that only diagnoses with the same or related meanings were marked as such. Otherwise, it returned “False”.

Similar methodologies have been explored successfully in recent research, showing that LLMs can effectively perform human-like evaluations in various tasks, including text summarization, quality assessments, and chat assistant evaluations, with results aligning closely to human judgments^43–45^. These findings support the use of LLMs as reliable tools for tasks like our diagnostic comparison evaluation.

Moreover, insights from the paper “Self-Recognition in Language Models”^46^ further argue that LLMs do not specifically prefer their own responses over those from other models. When asked to choose among several answers, the study showed that weaker models tend to select the one they consider as best, rather than their own, demonstrating that LLMs prioritize answer quality over origin. As a result, high-quality models are more likely to recognize their own outputs as good—not out of bias, but because of their focus on quality. This reinforces the idea that LLMs can perform evaluations without self-preference. Importantly, we did not use the LLM to compare outputs across models, which could risk introducing bias. Instead, the LLM evaluator compared each of the top three predicted diagnoses directly to the ground truth, determining whether they aligned in meaning or category. By focusing only on direct comparisons between predictions and the ground truth, we aimed to minimize self-bias and ensure an objective evaluation process.

While promising, the reliability and interpretability of LLMs as evaluation tools in real-world clinical environments still need further validation and refinement to ensure their safe and effective use. To address this, a subset of the dataset was validated by four clinicians, which is described in the subsection “Reader Study” in the “Methods” section. The results of this validation are detailed in the subsection “Inter-Rater Agreement on Diagnosis Evaluation” in the “Results” section.

### Reader study

In this study, we asked four clinicians from different institutions to review the performance of an LLM in generating and predicting clinical specialties and diagnoses. The clinicians come from diverse medical backgrounds, ensuring a broad perspective in the evaluation process with several years of experience. We included one clinician affiliated with Policlinico Gemelli in Rome, Italy, another with the Radiology Department at Klinikum rechts der Isar in Munich, Germany, and two clinicians are based at the University of Chicago in the United States.

The revision aimed to assess how well the LLM performed the following two tasks: first, creating a ground truth specialty based on the given diagnosis, and second, predicting diagnoses for each patient. We selected a subset of 400 out of the 2000 cases from the dataset. Each clinician was assigned 200 cases, with Clinicians 1 and 2 reviewing the same subset, and Clinicians 3 and 4 reviewing a different subset. This setup allowed for independent evaluations of the same cases by each pair, improving objectivity as much as possible.

For the first task, the clinicians evaluated the LLM generated ground truth specialty for each diagnosis. The clinicians assessed the accuracy of these predictions by categorizing them into the following four levels: Correct, where the prediction matched the specialty a clinician would select for the diagnosis; Partially Correct, where the prediction was relevant but not ideal, such as suggesting a generalist or related specialty; Reasonable but Suboptimal, where the prediction was valid but less optimal, demonstrating a plausible but less precise understanding of the diagnosis; and Incorrect, where the prediction had no logical connection to the diagnosis.

For the second task, we used a subset from the outputs of the clinical user setting of Claude 3.5 Sonnet and the RAG-assisted LLM. For each model the clinicians compared the LLM predicted diagnoses with the ground truth diagnoses and categorized them as follows: Exact Match, where the prediction matched the ground truth diagnosis exactly; Clinically Equivalent, where the prediction conveyed the same clinical condition as the ground truth but used slightly different terminology or scope; Clinically Related, where the prediction referred to a related condition relevant to clinical reasoning but diverged from the ground truth; and Incorrect, where the prediction was clinically unrelated to the ground truth.

The goal of this evaluation is to demonstrate that the LLM performs well in predicting both the specialty and the diagnosis, with a high level of acceptance among clinicians. In addition to predicting diagnoses, the LLM was also used to compare and evaluate these predicted diagnoses against the ground truth. Eventually add here that also here the clinicians review showed a well performance fo the llms.

This dual role highlights the LLM’s ability not only to generate outputs but also to assess its own performance. These findings show the potential of LLMs to assist in clinical decision-making and evaluation processes. By providing a cost-effective and time-efficient solution, LLMs could serve as a valuable tool to support clinicians and offer a reliable second opinion in medical practice.

### Intra-model agreement

We evaluated the agreement between models by comparing the predictions of different variants of the eight models, consisting of the RAG-assisted model and the three Claude language models with general user and clinical user settings each. Agreement was calculated separately for triage level predictions and specialty predictions and is symmetrical. Therefore, the results for both datasets are shown in the Supplementary Table 2, where the upper triangular matrix shows the intra-model agreement for triage and the lower triangular matrix for specialty, excluding self-comparisons (i.e., perfect agreement with the same model).

We evaluated and highlighted the two highest agreement values between model pairs for each dataset (specialty and triage) and for each of the three model user setting subgroups (general user to general user, general user to clinical user, clinical user to clinical user).

## Supplementary information

Supplementary information

## Data Availability

Core data is available at https://physionet.org/content/mimic-iv-note/2.2/. Data processing scripts and processed data are available at https://github.com/BIMSBbioinfo/medLLMbenchmark.

## Code availability

The code to process data is available at https://github.com/BIMSBbioinfo/medLLMbenchmark.
