# Supplementary material for: Evaluating large language model workflows in clinical decision support for triage and referral and diagnosis
Source: NPJ Digit Med. 2025 May 9;8:263. doi: 10.1038/s41746-025-01684-1 (PMC12064692; doi:10.1038/s41746-025-01684-1)
Supplement: Supplementary file 1 — Supplementary information [file 41746_2025_1684_MOESM1_ESM.pdf]

Supplementary Information

Evaluating large language model workflows in clinical decision support for triage and referral and diagnosis

A Prompts

You are a nurse with emergency and triage experience. Using the patient's history of present illness and his information, determine the triage level based on the Emergency Severity Index (ESI), ranging from ESI level 1 (highest acuity) to ESI level 5 (lowest acuity):
1: Assign if the patient requires immediate lifesaving intervention.
2: Assign if the patient is in a high-risk situation (e.g., confused, lethargic, disoriented, or experiencing severe pain/distress)
3: Assign if the patient requires two or more diagnostic or therapeutic interventions and their vital signs are within acceptable limits for non-urgent care.
4: Assign if the patient requires one diagnostic or therapeutic intervention (e.g., lab test, imaging, or EKG).
5: Assign if the patient does not require any diagnostic or therapeutic interventions beyond a physical exam (e.g., no labs, imaging, or wound care).

History of present illness: {HPI} and
patient info: {patient_info}.
Respond with the level in an <acuity> tag.

Supplementary Figure 1 | Triage level prediction prompt for the general user model. This prompt was used to generate triage level predictions using the general user model. The prompt instructs the LLM to act as a nurse with emergency and triage experience. The different triage levels are explained, and the LLM is asked to assign a triage level based on the provided input, which consists of the history of present illness and patient information. This approach reflects symptom-based data typically available to patients and excludes access to initial vital signs, aligning with the general user model approach.

You are a nurse with emergency and triage experience. Using the patient's history of present illness, his information and initial vitals, determine the triage level based on the Emergency Severity Index (ESI), ranging from ESI level 1 (highest acuity) to ESI level 5 (lowest acuity):
1: Assign if the patient requires immediate lifesaving intervention.
2: Assign if the patient is in a high-risk situation (e.g., confused, lethargic, disoriented, or experiencing severe pain/distress)
3: Assign if the patient requires two or more diagnostic or therapeutic interventions and their vital signs are within acceptable limits for non-urgent care.
4: Assign if the patient requires one diagnostic or therapeutic intervention (e.g., lab test, imaging, or EKG).
5: Assign if the patient does not require any diagnostic or therapeutic interventions beyond a physical exam (e.g., no labs, imaging, or wound care).

History of present illness: {HPI},
patient info: {patient_info} and
Initial vitals: {initial_vitals}.
Respond with the level in an <acuity> tag.

Supplementary Figure 2 | Triage level prediction prompt for the clinical user model. The shown prompt was used to generate triage level predictions using the clinical user model. The prompt instructs the LLM to act as a nurse with emergency and triage experience. The different triage levels are explained, and the LLM is asked to assign a triage level based on the provided input, which consists of the history of present illness, patient information and initial vitals. This approach reflects ED-based data typically available to clinicians and includes access to initial vital signs, aligning with the clinical user model approach.

You are an experienced healthcare professional with expertise in determining the medical specialty and diagnosis based on a patient's history of present illness and personal information. Review the data and identify the three most likely, distinct specialties to manage the condition, followed by the three most likely diagnoses.
List specialties first, in order of likelihood, then diagnoses.
Respond with the specialties in <specialty> tags and the diagnoses in <diagnosis> tags.
History of present illness: {hpi} and
personal information: {patient_info}.

Supplementary Figure 3 | Diagnosis and specialty prediction prompt for the general user model. The shown prompt was used for diagnosis and specialty prediction in the general user model. The prompt instructs the LLM to take on the role of an experienced healthcare professional. It assigns the task of determining the three most likely medical specialties the patient should visit and the three most probable diagnoses based on the provided input. In this general user model, the input is limited to the history of present illness and personal information, reflecting the typical data available to patients without access to clinical measurements or professional medical assessment.

You are an experienced healthcare professional with expertise in determining the medical specialty and diagnosis based on a patient's history of present illness, personal information and initial vitals. Review the data and identify the three most likely, distinct specialties to manage the condition, followed by the three most likely diagnoses.
List specialties first, in order of likelihood, then diagnoses.
Respond with the specialties in <specialty> tags and the diagnoses in <diagnosis> tags.
History of present illness: {hpi},
personal information: {patient_info} and
Initial vitals: {initial_vitals}.

Supplementary Figure 4 | Diagnosis and specialty prediction prompt for the clinical user model. The shown prompt was used for diagnosis and specialty prediction in the clinical user model. The prompt instructs the LLM to take on the role of an experienced healthcare professional. It assigns the task of determining the three most likely medical specialties the patient should visit and the three most probable diagnoses based on the

provided input. In this clinical user model, the input consists of the history of present illness, personal information and initial vitals. This reflects the typical data available in the ED.

> You are an experienced healthcare professional with expertise in medical and clinical domains. Determine the medical specialty most appropriate for the patient to consult based on the diagnosis. Please analyze the given diagnosis and predict the medical specialty that would typically manage the condition associated with it. If the condition might be treated by multiple specialties, prioritize the one most likely to manage the majority of cases.
> Respond with the specialty name only.
> Give the specialty in a <specialty> tag.
> If you can't find a specialty return 'no answer' in a <specialty> tag.
> Diagnosis: {diagnosis}.

Supplementary Figure 5 | Ground truth specialty assignment prompt. The shown prompt was used to create the specialty ground truth. The LLM is provided with a diagnosis and is instructed to act as an experienced healthcare professional. The prompt assigns the LLM the task to determine the appropriate medical specialty for the given diagnosis.

> You are an experienced healthcare professional with expertise in medical and clinical domains. I will provide a list of real diagnoses for a patient and 3 predicted diagnoses. For each predicted diagnosis, determine if it has the same meaning as one of the real diagnoses or if the prediction falls under a broader category of one of the real diagnoses (e.g., a specific condition falling under a general diagnosis category).
> If it matches, return 'True'; otherwise, return 'False'.
> Return only 'True' or 'False' for each predicted diagnosis within <evaluation> tags and nothing else.
> Real Diagnoses: {real_diag},
> predicted diagnosis 1: {diag1},
> predicted diagnosis 2: {diag2} and
> predicted diagnosis 3: {diag3}.

Supplementary Figure 6 | Diagnosis prediction evaluation prompt. The shown prompt was used for evaluating. The LLM is instructed to act as an experienced healthcare professional. For each patient, the prompt provides the LLM with a list of ground truth diagnoses and three predicted diagnoses. The LLM is tasked with determining, for each predicted diagnosis, whether it is included in the list of ground truth diagnoses or if it falls under a broader category of one of the ground truth diagnoses.

B Emergency Severity Index

ESI Level	Description
Level 1	It is assigned to patients requiring immediate life-saving interventions, such as those in cardiac arrest or severe respiratory distress.
Level 2	It includes patients in high-risk situations, such as those who are confused, lethargic, or in severe pain, requiring urgent medical attention.
Level 3	It applies to patients who need two or more diagnostic or therapeutic interventions, but whose vital signs are stable enough that immediate intervention is not critical.
Level 4	It is for patients requiring only one diagnostic or therapeutic intervention, such as a lab test or imaging study.
Level 5	It is used for patients who do not require any diagnostic or therapeutic interventions beyond a physical exam, such as those presenting with minor complaints.

Supplementary Table 1 | Emergency Severity Index (ESI) description. This table shows the five levels of the Emergency Severity Index (ESI) system. The urgency and the typical characteristics associated with each level are described.

C Specialty Distribution

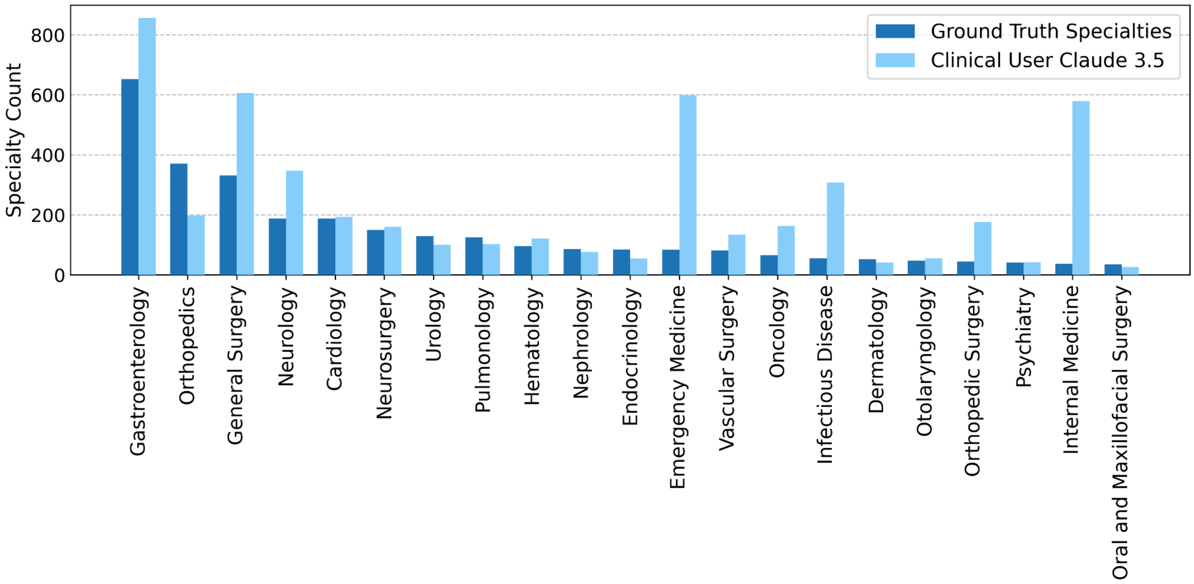

Supplementary Figure 7 | Specialty distribution: ground truth vs. prediction by Claude 3.5 Sonnet. This graph illustrates the ground truth quantities of the most frequent medical specialties with their predicted specialty quantities of the best-performing model, Claude 3.5 Sonnet.

D Intra-model agreement

Model		General User				Clinical User			
		RAG-Assisted LLM	Claude 3.5 Sonnet	Claude 3 Sonnet	Claude 3 Haiku	RAG-Assisted LLM	Claude 3.5 Sonnet	Claude 3 Sonnet	Claude 3 Haiku
General User	RAG-Assisted LLM	-	**81,60**	<u>73,40</u>	71,60	<u>85,05</u>	79,35	75,75	69,30
	Claude 3.5 Sonnet	**85,62**	-	72,70	70,00	78,20	**86,35**	74,25	62,10
	Claude 3 Sonnet	77,48	<u>79,87</u>	-	70,65	73,25	70,65	77,90	67,60
	Claude 3 Haiku	76,53	79,07	79,53	-	71,40	68,55	72,65	75,00
Clinical User	RAG-Assisted LLM	86,82	85,85	77,15	76,35	-	**82,35**	<u>78,50</u>	72,55
	Claude 3.5 Sonnet	84,53	**93,12**	79,57	79,03	**85,95**	-	76,85	64,55
	Claude 3 Sonnet	76,30	79,05	92,00	79,25	77,18	79,32	-	72,30
	Claude 3 Haiku	76,67	79,30	79,58	<u>92,07</u>	76,67	79,78	<u>80,10</u>	-

Supplementary Table 2 | Inter-model agreement matrix for triage level and specialty predictions. Upper triangular matrix shows the inter-model agreement [%] for triage level and lower triangular matrix for specialty. Bold marks the highest agreement in each model type group (general user to general user, general user to clinical user, and clinical user to clinical user) and underlined the second highest. Agreement to the same model is omitted, as it is a complete agreement

E Clinician Validation of LLM-Generated Specialties

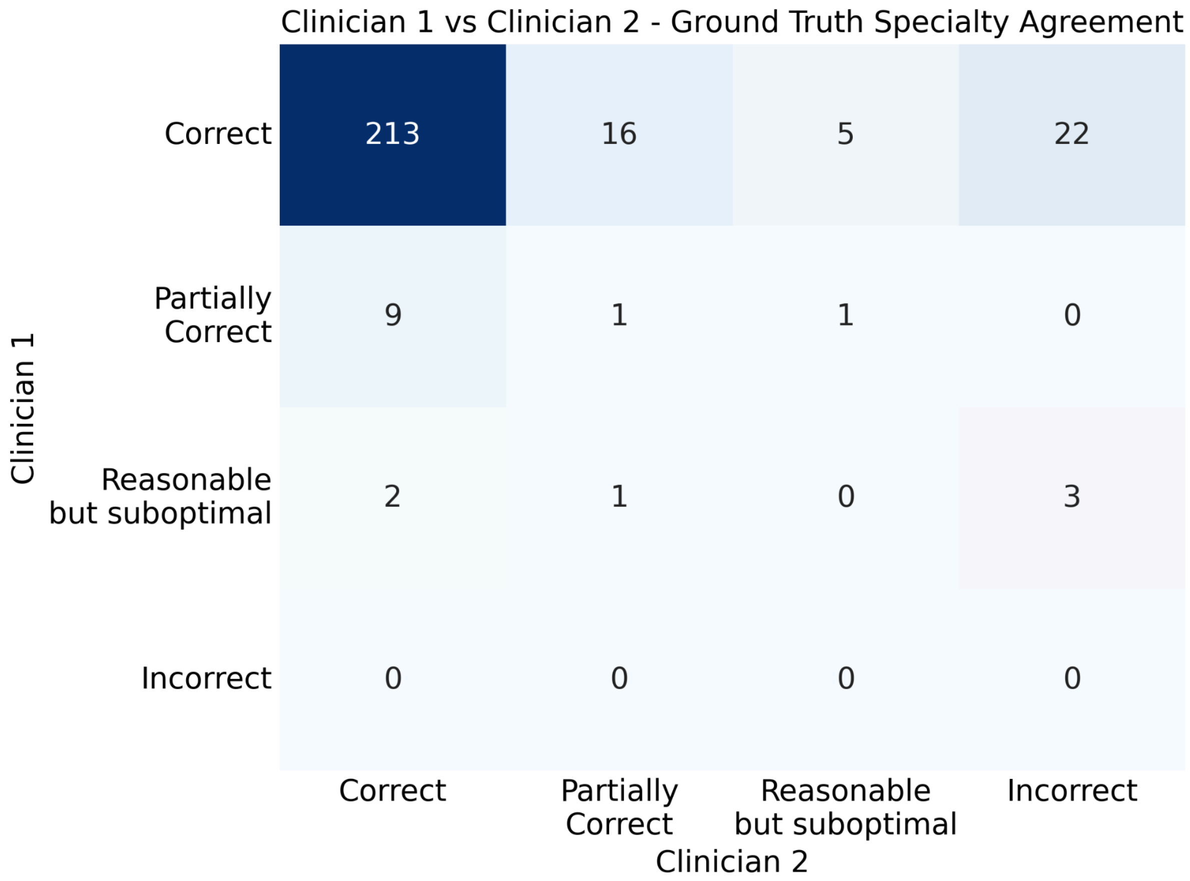

Supplementary Figure 8 | Inter-rater agreement (Clinician 1 and 2) on the LLM-generated ground truth specialties. This confusion matrix visualizes the agreement between Clinician 1 and Clinician 2 in their review of the ground truth specialties generated by the LLM. The matrix provides an overview of how often the two clinicians agreed or differed in their review of the LLM's specialty assignments.

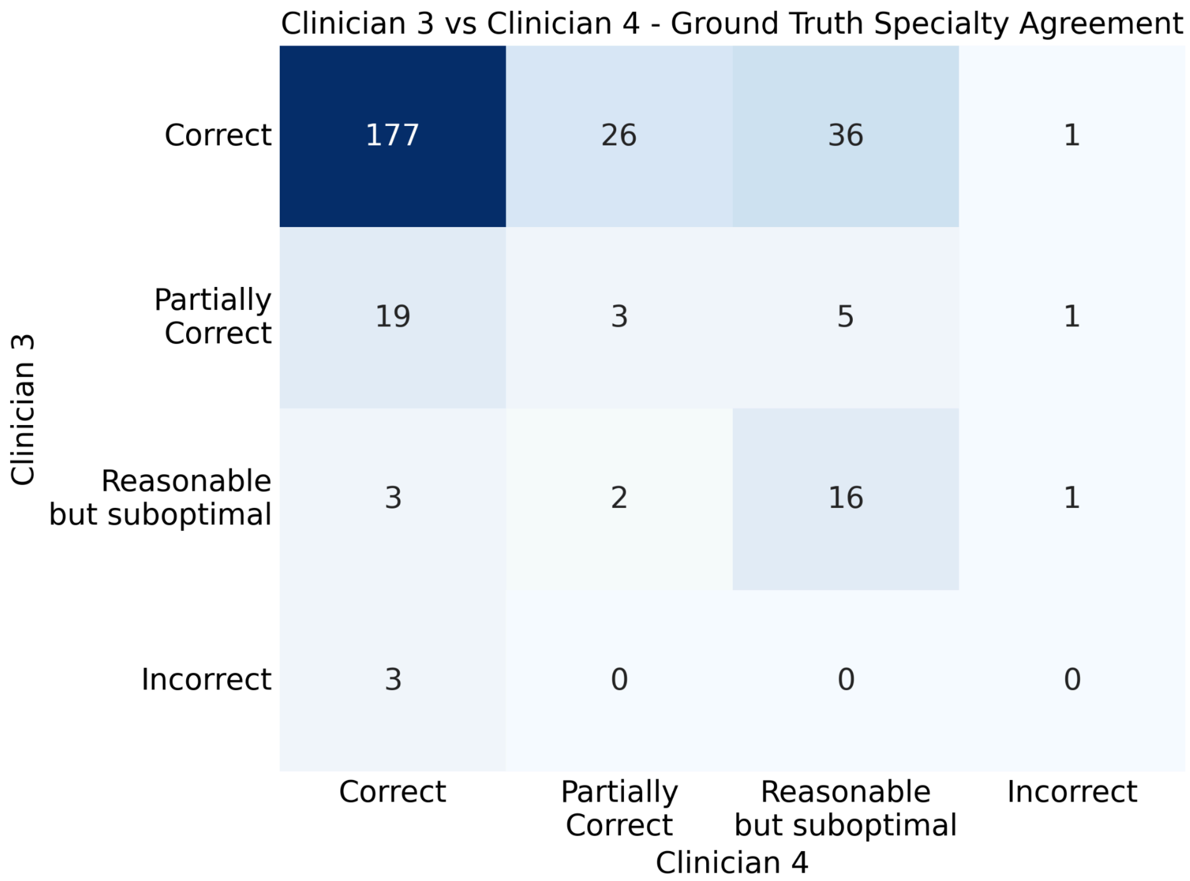

Supplementary Figure 9 | Inter-rater agreement (Clinician 3 and 4) on the LLM-generated ground truth specialties. This confusion matrix visualizes the agreement between clinician 3 and Clinician 4 in their review of the ground truth specialties generated by the LLM. The matrix provides an overview of how often the two clinicians agreed or differed in their review of the LLM's specialty assignments.

F Inter-Rater-Agreement on Diagnosis Evaluation

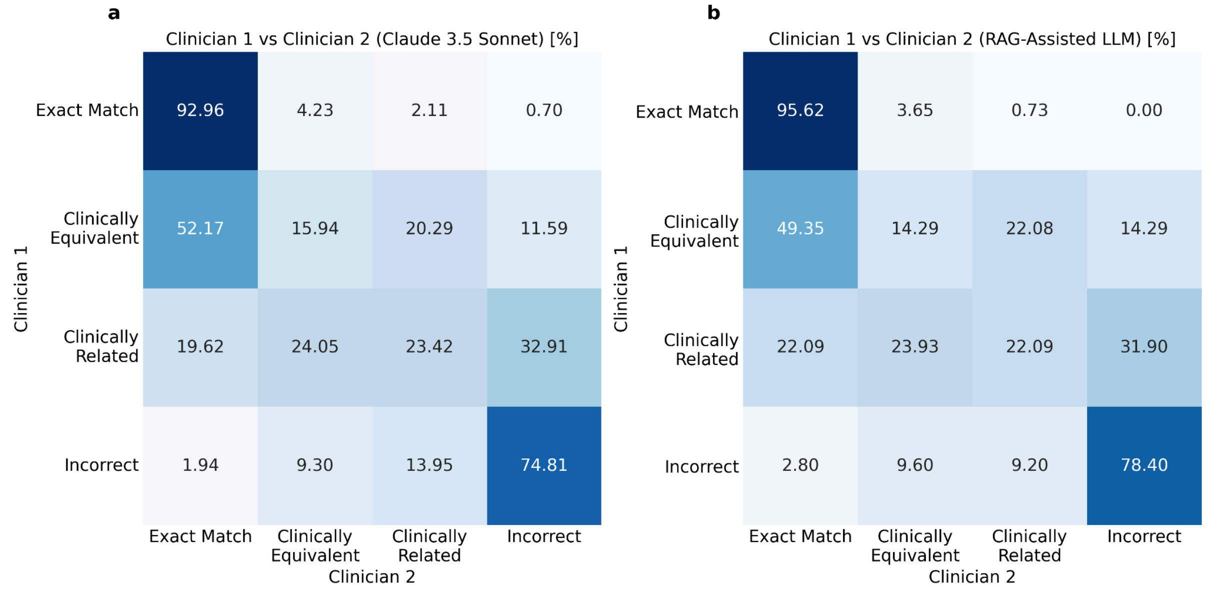

Supplementary Figure 10 | Inter-rater agreement (clinician 1 and 2) on the predicted vs. ground truth diagnoses. These confusion matrices visualize the agreement between clinician 1 and Clinician 2 in their review of the predicted diagnoses compared to ground truth diagnoses. The matrices provide an overview of how often the two clinicians agreed or differed in their assessment of the diagnosis prediction for **a** Claude 3.5 Sonnet and **b** RAG-Assisted LLM. This comparison shows the discrepancies and agreements in the clinicians' judgments of the diagnosis predictions

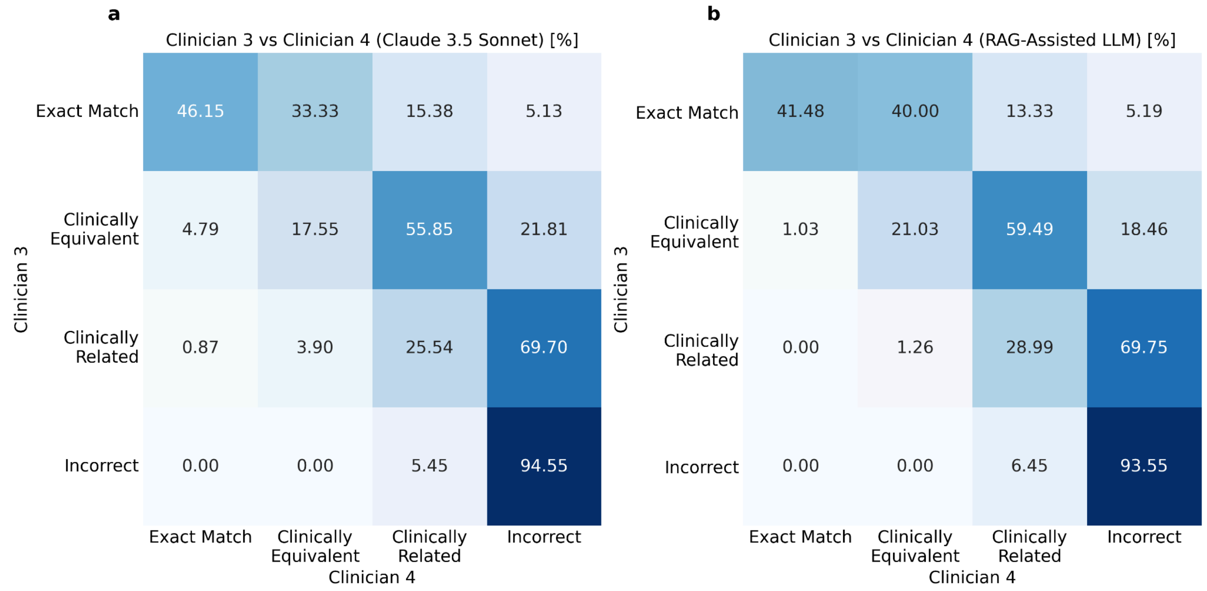

Supplementary Figure 11 | Inter-rater agreement (clinician 3 and 4) on the predicted vs. ground truth diagnoses. These confusion matrices visualize the agreement between Clinician 3 and Clinician 4 in their review of the predicted diagnoses compared to ground truth diagnoses. The matrices provide an overview of how often the two clinicians agreed or differed in their assessment of the diagnosis prediction for **a** Claude 3.5 Sonnet and **b** RAG-Assisted LLM. This comparison shows the discrepancies and agreements in the clinicians' judgments of the diagnosis predictions
